# Feasibility and effectiveness of prone position in morbidly obese ARDS patients: a case-control clinical study

**DOI:** 10.1186/cc12054

**Published:** 2013-03-19

**Authors:** A De Jong, N Molinari, M Sebbane, A Prades, S Jaber

**Affiliations:** 1 Montpellier University Hospital, Montpellier, France

## Introduction

Obese patients are at risk of developing atelectasis and acute respiratory distress syndrome (ARDS) [1]. The prone position (PP) may reduce atelectasis, and improves oxygenation and outcome in severe hypoxemic patients in ARDS [2], but little is known about its effect in obese ARDS patients.

## Methods

Morbidly obese patients (body mass index (BMI) ≥35 kg/m^2^) in PP with ARDS (PaO_2_/FiO_2 _ratio ≤200 mmHg) were matched to nonobese (BMI <30 kg/m^2^) ARDS patients in a case-control clinical study. The primary endpoints were safety and complications of PP; the second endpoints were the effect on oxygenation (PaO_2_/FiO_2 _ratio at the end of PP), length of mechanical ventilation and ICU stay, nosocomial infections and mortality.

## Results

**Figure 1:**
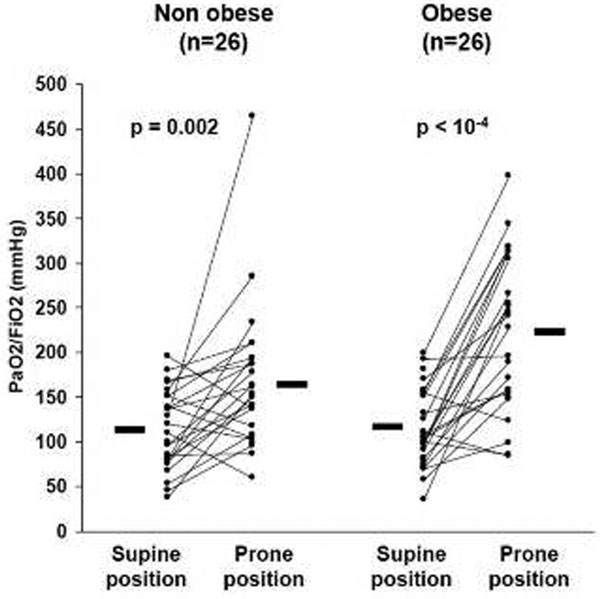
**Individual variations of PaO_2_/FiO_2 _ratio between supine and prone positions in obese and nonobese patients**.

Between January 2005 and December 2009, 149 patients were admitted for ARDS. Thirty-three obese patients were matched with 33 nonobese patients. Median PP duration was 9 (6 to 11) hours in obese patients and 8 (7 to 12) hours in nonobese patients (*P *= 0.28). We collected 51 complications, of which 25 in obese patients and 26 in nonobese patients. The number of patients with at least one complication was similar across groups (*n *= 10, 30%). The PaO_2_/FiO_2 _ratio (Figure 1) increased significantly more in obese patients (from 118 ± 43 to 222 ± 84 mmHg) than in nonobese patients (from 113 ± 43 mmHg to 174 ± 80 mmHg, *P *= 0.03). Length of mechanical ventilation, ICU stay and nosocomial infections did not differ significantly, but mortality at 90 days was significantly lower in obese patients (27 vs. 48%, *P <*0.05).

## Conclusion

PP seems safe in obese patients and may improve oxygenation more than in nonobese patients. Obese patients could be a subgroup of ARDS patients who may benefit most from PP.
